# FlowT-SR: A Novel Remote Sensing Image Super-Resolution Framework with Cloud Haze and Noise Suppression

**DOI:** 10.3390/s26165094

**Published:** 2026-08-11

**Authors:** Yutong Zhang, Guang Yang, Rongxiang Liu, Yuebao Wang, Xiaotong Guo

**Affiliations:** 1 Software Engineering Technology Research Center, University of Emergency Management, Sanhe 065201, China; zytncist2026@163.com (Y.Z.); 25661626@st.cidp.edu.cn (R.L.); b1296526160@163.com (Y.W.); guoxiaotong2024@163.com (X.G.); 2 Hebei Province University Smart Emergency Application Technology Research and Development Center, Langfang 065201, China

**Keywords:** super-resolution, interference suppression, denoising, cloud removal, flow matching, diffusion transformer

## Abstract

Remote sensing image super-resolution (SR) aims to enhance spatial resolution and recover image details, which typically enhances the quality of optical remote sensing imagery. However, interference from cloud haze cover and sensor noise often leads to distorted details and artifacts in reconstructed images of conventional deep learning SR approaches, significantly limiting reconstruction fidelity. To address these challenges, we propose a novel SR framework based on the flow matching paradigm and a diffusion transformer, named FlowT-SR, which achieves superior and reliable reconstruction quality by jointly mitigating sensor noise and thin cloud interference. First, an evolution path from low-resolution images to ground-truth images is constructed based on the optimal transport displacement interpolation mechanism, and the corresponding vector field that governs this evolution is employed as the supervision signal for subsequent model training. Then, a multi-scale interference suppression (MSIS) module is combined with a novel diffusion transformer network (DiTNet) to predict the vector field. The MSIS module performs preliminary denoising and captures the spatial distribution of thin cloud and haze in low-resolution images, providing degradation-aware feature representations for DiTNet. Subsequently, a DiTNet is presented to predict the evolution vector field obtained in the first stage, which consists of ten layers based on the diffusion transformer. By accurately predicting the vector field at any time step, the model effectively reduces the impact of cloud haze and noise interference to improve the reconstruction precision. Finally, driven by the predicted vector field along the evolution path, the SR remote sensing image is generated through solving the corresponding ordinary differential equation, yielding cloud-free and noise-reduced results. Extensive experiments on our dataset and the public CUHK Cloud Removal dataset demonstrate that FlowT-SR effectively suppresses cloud haze and noise interference, achieving superior reconstruction performance compared with current state-of-the-art methods in terms of both PSNR and SSIM.

## 1. Introduction

High-resolution (HR) optical remote sensing images provide abundant and reliable information on ground features for urban development, risk assessment, and post-disaster evaluation [1,2]. However, extracting fine structural details from optical remote sensing images remains a significant challenge due to the inherent limitations of spatial resolution and adverse imaging conditions [3]. Consequently, super-resolution (SR) technology, which aims to improve the resolution and quality of low-resolution (LR) optical remote sensing images, has become a prominent research topic in remote sensing image processing [4,5].

In recent years, with the rapid advancement of satellite imaging technology and deep learning methods, many researchers have explored the application of deep generative models for optical remote sensing image SR to boost data quality and practical applications [6,7,8]. There are generally four categories of methods: convolutional neural network (CNN)-based, generative adversarial network (GAN)-based, flow-based, transformer-based and diffusion-based approaches [9,10].

Tian et al. [11] proposed a coarse-to-fine CNN that integrates global and local contextual information to enhance fine-grained structural details, improving texture clarity and reconstruction fidelity. This method achieves reasonable performance in recovering textures in grassland areas. However, due to the limited receptive field of convolutional operators, reconstructed images may exhibit artifacts in buildings and roads, such as jagged boundaries and contour distortions.

GANs are capable of capturing complex data distributions and generating results that are closer to real samples. Therefore, they are widely used in SR tasks. Dong et al. [12] proposed a GAN-based SR model that effectively leverages information from HR reference images, which improves the reconstruction of edge details. Jia et al. proposed a multi-attention GAN that incorporates pyramid convolution residual dense blocks, attention up-sampling and attention fusion to achieve high-resolution remote sensing image generation [13]. Zhang et al. proposed an edge-enhanced and structure-aware GAN for remote sensing images and utilized an edge-guided loss to enhance image sharpness and perceptual consistency [14]. However, these methods are limited by the difficulty of training the generator, and the generated results may exhibit instability.

Lugmayr et al. [15] proposed SRFlow, a normalizing flow-based SR model that learns the conditional distribution of HR images via a conditional normalizing flow architecture, enabling realistic HR reconstruction. Yao et al. [16] introduced Local Implicit Normalizing Flow, which allows the generation of HR images with rich texture details at arbitrary upscaling factors. Song et al. [17] proposed a wavelet-domain normalizing flow framework, in which a refinement module is employed to enhance the recovery of image details. However, traditional flow-based methods are often limited by high computational complexity and relatively slow inference due to the requirement of invertible architectures and the computation of Jacobian determinants [18,19].

SwinIR pioneered the application of the Swin Transformer to image restoration, demonstrating superior reconstruction quality and parameter efficiency over conventional CNN-based methods [20]. Building upon SwinIR, several task-specific variants have been proposed to improve robustness under real-world degradations [21,22,23]. HiT-SR adopts a hierarchical transformer architecture with expanding window attention and efficient spatial-channel correlation, reducing computational complexity while maintaining high reconstruction accuracy [24]. In addition, ViSIR integrates Vision Transformers with Sinusoidal Representation Networks to mitigate transformer spectral bias and improve high-frequency detail reconstruction [25]. Transformer-based SR methods have good performance by leveraging self-attention mechanisms to capture long-range dependencies and global contextual information. However, they generally have limited capability in preserving fine local textures, motivating the development of more efficient hybrid architectures that combine transformers with complementary feature extraction techniques.

Saharia et al. [26] proposed SR3, which applies diffusion models to the SR task. Starting from pure noise, the model exploits a U-Net architecture to iteratively predict and remove noise, ultimately reconstructing an HR image. Compared with GAN-based and flow-based approaches, SR3 achieves more stable training and demonstrates superior performance in reconstructing large contiguous forests and farmland regions. Xiao et al. [27] proposed EDiffSR, which first extracts and enhances effective information from LR images to guide the restoration of image details and structures. However, the generated images may still exhibit limited continuity and structural inconsistencies in building edges, roof contours, and road boundaries. StableSR utilizes the prior knowledge from large pre-trained diffusion models [28]. This method adaptively adjusts the strength of conditional guidance at different denoising steps, reducing the influence of irrelevant information from LR images. However, this method still requires further improvement in preserving the coherence of large-scale structures. Miao et al. proposed a cross-sensor SR method based on diffusion models and designed a prior feature extraction module and an efficient sampling strategy to utilize LR priors for HR reconstruction while reducing computational costs [29]. DiT4SR is proposed based on the diffusion transformer (DiT) [30]. It extracts latent representations from LR images and feeds them into a self-attention-based noise prediction network, enhancing the model’s ability to capture long-range dependencies and enabling more coherent reconstruction of large-scale structural boundaries. Although numerous studies have explored diffusion-based SR methods due to their ability to generate diverse samples, these approaches typically require a large number of iterative sampling steps, resulting in high computational cost and slow generation speed, which constrains their practical efficiency [31,32].

Lipman et al. proposed a generative framework, flow matching, which transforms the nonlinear mapping between images into a conditional probability path estimation problem from Gaussian noise to the target image conditioned on LR images. Moreover, a U-Net model predicts an approximately linear path, improving generation efficiency while preserving the realism of the generated images [33]. Gong et al. [34] proposed SfmSR based on flow matching for remote sensing image SR. The method first leverages pretrained weights to learn multi-scale representations of remote sensing images. It then guides the flow matching generation process using LR images and their corresponding semantic information. This approach enables efficient image generation with a relatively small number of parameters.

Above mentioned approaches have achieved notable progress in SR tasks. However, cloud, haze and sensor noise in LR images severely affect the semantic consistency between generated and real images. Feng et al. proposed a RRDGAN model based on GAN which incorporates denoising and SR into a unified framework to improve the generation quality [35]. Moreover, in the process of cloud removal, SR methods are often utilized to construct datasets, and integrating multiple tasks into a single model to simplify the calculation and processing procedures has become a trend [36,37]. However, the existing methods are still difficult to accurately suppress the interference from clouds, haze and sensor noise, particularly when images are degraded by cloud haze occlusion and sensor noise that obscure object boundaries and other critical details.

Therefore, we propose a novel anti-interference super-resolution framework for optical remote sensing images with cloud haze and noise suppression based on flow matching and a diffusion transformer, named FlowT-SR. Instead of relying on the conventional image-to-image generation processes, FlowT-SR predicts the vector field within the flow matching framework, enabling high-quality and realistic SR reconstruction with only a few sampling steps, thereby substantially reducing the reconstruction cost. Moreover, we design a multi-scale interference suppression (MSIS) module to conduct preliminary noise suppression and capture cloud–haze spatial patterns in low-resolution images, providing more reliable conditional information for subsequent vector field prediction. Furthermore, we design a vector field prediction network (DiTNet) consisting of ten diffusion transformer blocks (DiTBlocks). By leveraging the global modeling capability of the attention mechanism, DiTNet captures critical long-range structural dependencies and detailed texture information while reducing the influence of cloud haze interference. FlowT-SR not only improves computational efficiency but also effectively mitigates noise and cloud haze artifacts, producing more realistic super-resolved images.

The main contributions of this work are summarized as follows:

1. FlowT-SR is a novel unified framework that simultaneously performs super-resolution, cloud removal, and denoising, eliminating the need for separate restoration pipelines. By integrating the flow matching paradigm, the MSIS module, and DiTNet, the proposed method not only enhances reconstruction accuracy and fidelity but also significantly reduces the iterative reconstruction process through efficient sampling and feature modeling.
2. The MSIS module is designed to initially suppress noise, capture thin cloud–haze-related spatial patterns, and enhance spectral signatures in LR images, providing more effective information for the subsequent model learning and prediction.
3. The DiTNet is introduced to predict the vector field to improve reconstruction accuracy while maintaining texture fidelity and global structural consistency, and to suppress degradations caused by cloud haze and noise in low-resolution (LR) images.

## 2. Materials and Methods

**Figure 1:**
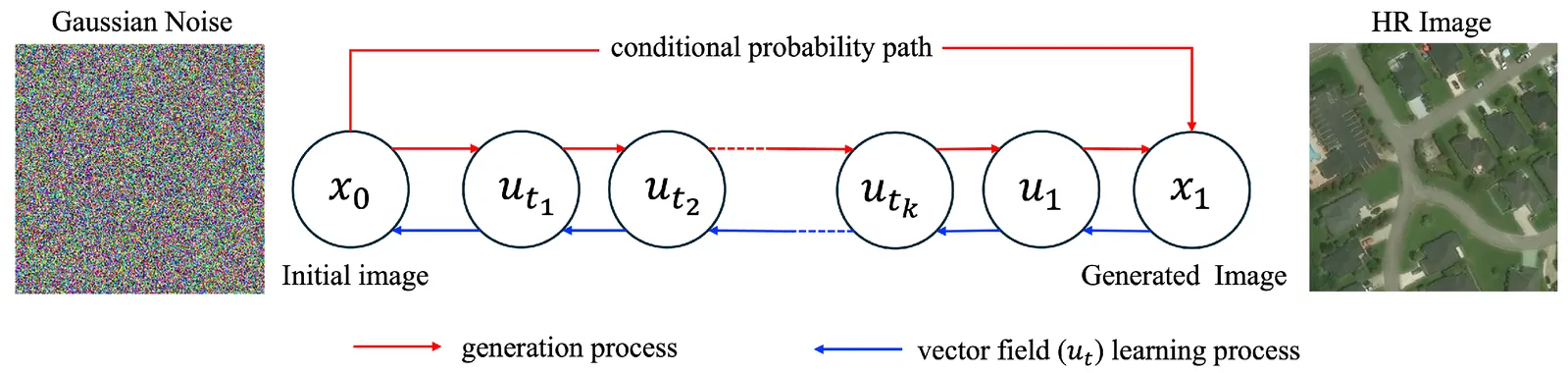
Schematic illustration of the flow matching process. The initial image represents noise and the generated image represents the final output of the model.

Flow matching is a generative modeling framework that learns a conditional probability path for image generation [33]. As illustrated in Figure 1, it defines a continuous-time evolution process t∈[0,1], which transports Gaussian noise to the ground-truth (GT) image. This process is governed by a time-dependent vector field $u_{t}$, which determines the direction of sample evolution at time t∈[0,1].

**Figure 2:**
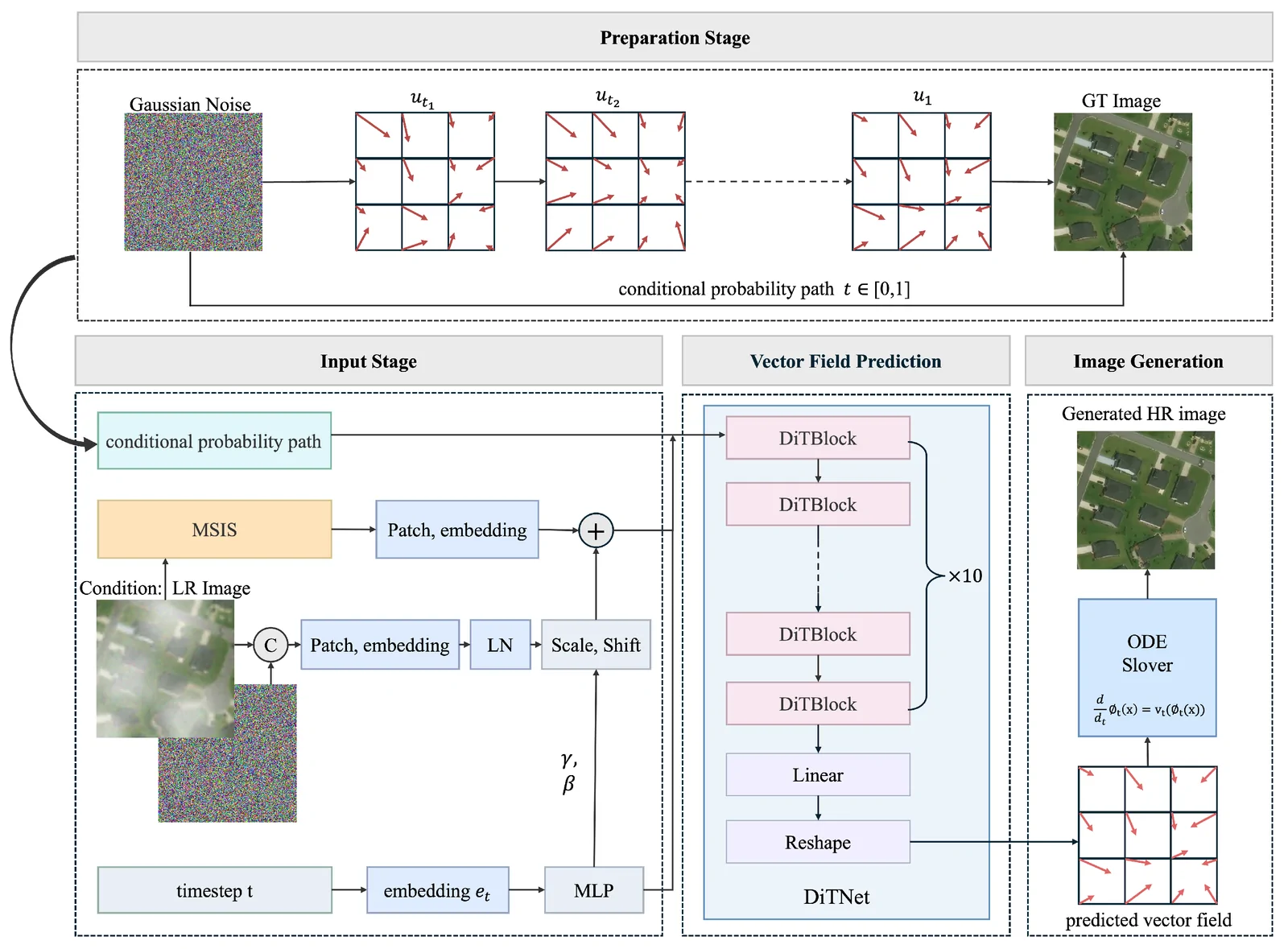
The architecture of our FlowT-SR.

Our FlowT-SR aims to generate realistic and reliable images while suppressing interference from thin cloud haze and noise. Based on the flow matching framework, our method is divided into four stages, as shown in Figure 2.

In the preparation stage, a conditional probability path between Gaussian noise and HR images is constructed based on optimal transport displacement interpolation mechanism, and the time-dependent vector field $u_{t}$ is computed along this path at any time step t∈[0,1]. In particular, the vector field is employed as the learning target during training to reduce the computational complexity.

During the input stage, the multi-scale interference suppression (MSIS) module is designed to initially suppress noise and capture the spatial distribution of thin cloud haze for the LR image. The feature maps extracted by MSIS are fused with a Gaussian noise image as the model input.

In the prediction stage, we design a DiTNet to learn vector fields based on a diffusion transformer (DiT), which consists of 10 DiTBlocks and leverages multi-head attention to capture global dependencies. A time-adaptive modulation mechanism dynamically adjusts features according to the time step *t*, enabling accurate prediction of the vector field along the conditional probability path at different time steps.

Finally, the predicted vector fields are utilized to generate the HR image by solving the ordinary differential equation (ODE) using an Euler solver. FlowT-SR reformulates the conventional nonlinear image generation problem into a linear evolution trajectory from LR images to HR images, which simplifies model prediction. Moreover, the MSIS module and DiTNet effectively mitigate interference during prediction, improving the realism of generated images and the stability of the generation process.

### 2.1. Preparation Stage

The time-dependent vector field ($u_{t}$) is estimated based on the optimal transport displacement interpolation mechanism to govern a continuous-time evolution path (ψ) from LR images ($x_{0}$) to GT images ($x_{1}$), as described below.

(1) $$x_{1}=\psi \left(x_{0}\right),$$

(2) $$x_{t}=\psi_{t}\left(x_{0}\right)=\left(1-t\right)x_{0}+t\psi \left(x_{0}\right)=\left(1-t\right)x_{0}+tx_{1},$$

(3) $$u_{t}=\frac{dx_{t}}{dt}=\psi \left(x_{0}\right)-x_{0}=x_{1}-x_{0}.$$

where t∈[0,1] denotes the time step in the generation process, $x_{t}$ represents a random variable sampled at time *t*, and $\psi_{t}$ represents a predicting evolution path at time *t*.

Hence, the conventional nonlinear image super-resolution problem is reformulated as a linear evolution trajectory estimation task, thereby significantly reducing the computational complexity.

### 2.2. Input Stage

In order to achieve better input effectiveness and ensure accurate subsequent learning, a multi-scale interference suppression (MSIS) module is designed to initially suppress the degradations of sensor noise and capture the spatial distribution of thin clouds and fog in the LR images.

**Figure 3:**
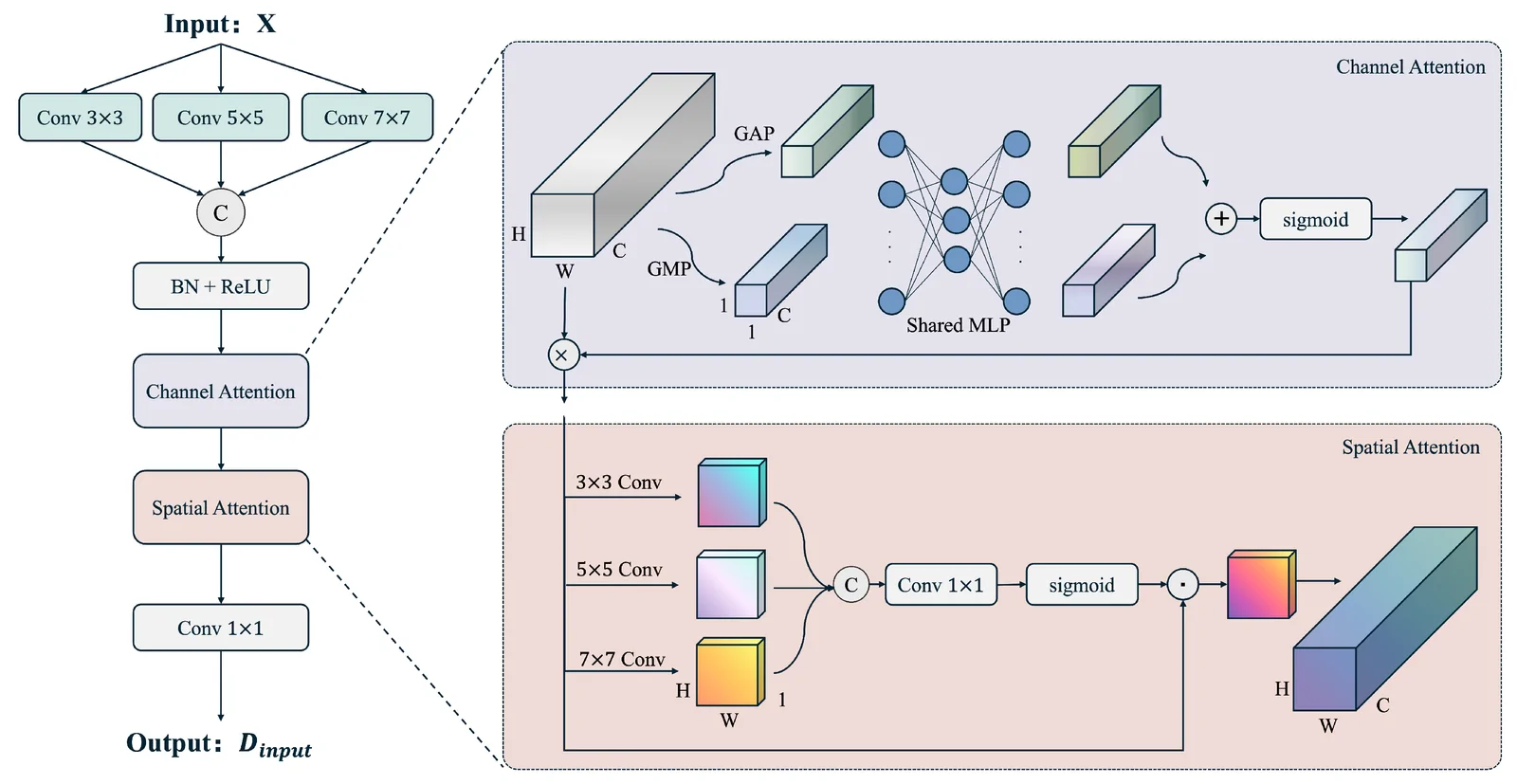
Detailed architecture of the MSIS module.

As shown in Figure 3, the MSIS module is designed to enhance the representation of degradation-related information in low-resolution (LR) images through multi-scale feature extraction and attention-based refinement. Specifically, multi-scale convolutions are first employed to capture structural information from different receptive fields, enabling robust feature extraction and reducing the impact of noise artifacts by leveraging complementary contextual information. Subsequently, channel and spatial attention mechanisms are introduced to adaptively emphasize informative features. The channel attention module enhances discriminative spectral characteristics, while the spatial attention module focuses on regions with potential thin cloud and haze interference, providing cloud-aware feature representations for subsequent reconstruction. Finally, the refined features are aggregated through a 1×1 convolution and embedded as input tokens. Through this design, MSIS implicitly learns noise suppression and thin cloud localization capabilities from the training data, improving the quality of degradation-aware feature representations for super-resolution reconstruction.

In addition, the original feature representation $f_{o}$ is obtained by merging the LR image with Gaussian noise, then partitioning it into non-overlapping local patches, embedding each patch into a high-dimensional feature space, and finally applying layer normalization (LN). The encoded timestep $e_{t}$ is fed into the MLP composed of multiple linear transformations and nonlinear activation functions to generate feature modulation parameters, namely the scale (γ) and shift (β). The parameter γ is generated to adaptively rescale the feature responses of individual channels in $f_{o}$, enabling the model to emphasize more relevant features at each time step. Meanwhile, β acts as a global bias term that shifts the feature representations, effectively regulating the distribution of feature activations. Finally, the input $D_{\mathrm{input}}$ for the vector field prediction is given by:

(4) $$D_{\mathrm{input}}=F_{m}⊕\left[\gamma ⊙f_{o}+\beta \right]$$

(5) $$\left[\gamma ,\beta \right]=w_{1}h+b_{1}$$

(6) $$h=f\left(w_{2}e_{t}+b_{2}\right)$$

where f(·) denotes the SiLU (Sigmoid Linear Unit) activation function, *h* denotes the feature map obtained by applying the MLP to $e_{t}$, and $w_{1},w_{2},b_{1},b_{2}$ are learnable parameters.

### 2.3. Vector Field Prediction with DiTNet

To capture global spatial dependencies, reduce the interference from noise and thin clouds, and improve the precision of vector field prediction at any time step t∈[0,1], this study designs a novel DiTNet based on a diffusion transformer, as shown in Figure 2. DiTNet consists of 10 stacked DiTBlocks, followed by an output projection layer to enhance its ability to model long-range dependencies.

**Figure 4:**
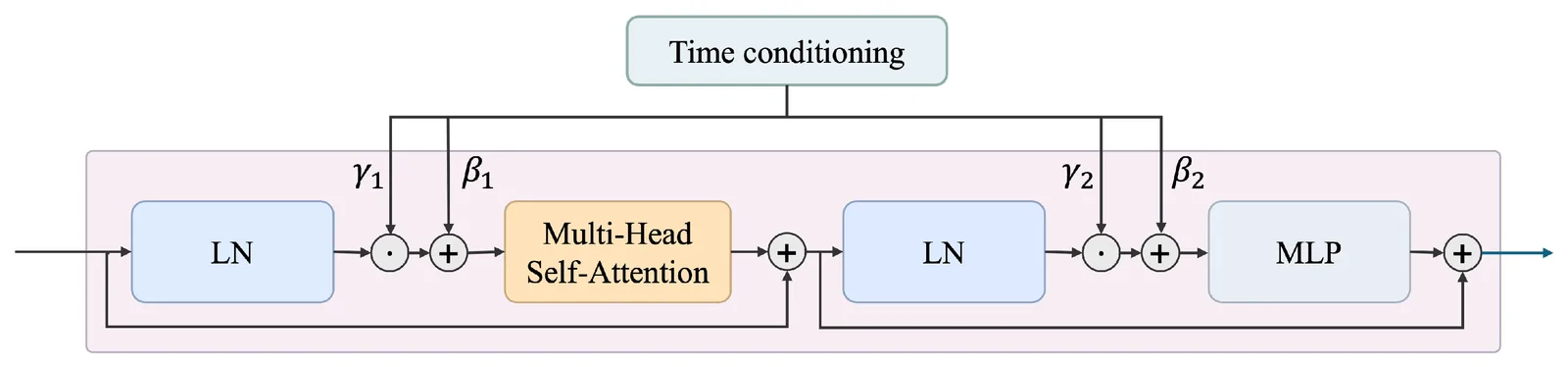
Detailed architecture of the DiTBlock.

The architecture of the DiTBlock is shown in Figure 4. The input tokens are first normalized using LN and their features are then modulated according to the current time step:

(7) $$F_{1}=\gamma_{1}⊙\mathrm{LN}\left(D_{\mathrm{input}}\right)+\beta_{1}.$$

Next, a multi-head attention (MHA) module is applied to model dependencies among tokens through multiple parallel attention heads, capturing long-range relationships in remote sensing images across different feature subspaces. The resulting features are combined with the original input via a residual connection, yielding H. After passing through another LN conditioned on time step

(8) $$F_{2}=\gamma_{2}⊙\mathrm{LN}\left(H\right)+\beta_{2}.$$

The parameters $\gamma_{1}$, $\beta_{1}$, $\gamma_{2}$, and $\beta_{2}$ are generated following the same mechanism used for the previously introduced γ and β parameters. We apply an MLP to perform element-wise nonlinear transformations on each token, which enhance the representation of local structures and details. Finally, the MLP output is combined with the attention branch through a residual connection, further refining and strengthening the representation of structural and detailed information.

Through repeated reconstruction and refinement of image representations, DiTNet maintains the structural coherence of remote sensing imagery. As a result, it can accurately capture the global layout and scene structure under challenging scenarios such as thin cloud haze occlusion, noise perturbation, or texture discontinuities. With continuous reconstruction, correction, and enhancement of features in deep layers, damaged structural regions can be progressively restored, as the 10-layer structure provides more rounds of optimization and improves the feature refinement capability. Ultimately, DiTNet adopts a two-step output reconstruction strategy, consisting of linear projection followed by a reshape operation, to convert token representations back into the complete vector field. This strategy mitigates false edges and detail distortions that frequently occur under cloud haze and noise interference by reconstructing a complete vector field from the structural and detailed information learned by the DiTBlocks through DiTNet.

The global dependency modeling capability of DiTNet is well suited for remote sensing scenarios that require stable reconstruction of large-scale structures and cross-region textures. It maintains stable reconstruction performance even under complex conditions such as noise interference and cloud haze occlusion.

The training objective is to make the predicted vector field approximate the GT vector field. This is achieved by minimizing the mean squared error between them:

(9) $$L_{\mathrm{CFM}}\left(\theta \right)=E_{t}{∥v_{t}-u_{t}∥}^{2}$$

where $v_{t}$ is the vector field predicted by DiTNet, and $u_{t}$ denotes the target vector field.

### 2.4. Image Generation Stage

By accumulating the changes at each step from the initial time to the final time, the final image is obtained as follows:

(10) $$x_{t}=x_{0}+\int_{0}^{t}u_{t}\;d_{t}$$

An ordinary differential equation (ODE) Euler solver is employed to estimate the generated image due to its simplicity, low computational cost, and fast inference speed compared with higher-order ODE solvers, providing an effective balance between reconstruction quality and sampling efficiency. The formulation is defined as:

(11) $$x_{t}=x_{t-1}+u_{t}\times \Delta t$$

where Δt represents the step size between two consecutive updates and controls the magnitude of each update.

As a result, the difficulty and error magnitude of each prediction step are reduced by approximating the evolution path as a linear trajectory. FlowT-SR can complete the generation process with fewer steps while maintaining high generation quality. Consequently, error accumulation during generation is effectively suppressed, which improves inference efficiency and enables more stable recovery of high-frequency textures and structural details in remote sensing images. Additionally, it reduces artifacts such as blurred details and false edges caused by thin cloud haze occlusion and noise interference.

### 2.5. Evaluation Metrics

To comprehensively evaluate the quality of the generated images, we employ three metrics: Peak Signal-to-Noise Ratio (PSNR), Structural Similarity Index (SSIM) [38], and Learned Perceptual Image Patch Similarity (LPIPS) [39].

#### 2.5.1. Peak Signal-to-Noise Ratio (PSNR)

The PSNR is employed to measure pixel-level reconstruction fidelity and is calculated based on the mean squared error (MSE) between the generated image and the reference image as follows. A higher PSNR indicates a smaller pixel-level difference between the generated image and the GT image, and the overall brightness and contrast of the two images are more similar.

(12) $$\mathrm{PSNR}=10\cdot \log_{10}\left(\frac{MAX^{2}}{\mathrm{MSE}}\right)$$

The MSE is defined as:

(13) $$\mathrm{MSE}=\frac{1}{mn}\sum_{i=0}^{m-1}\sum_{j=0}^{n-1}{\left[I(i,j)-K(i,j)\right]}^{2}$$

where MAX represents the maximum possible pixel value of the image, I(i,j) denotes the pixel value of the generated image at position (i,j), and K(i,j) denotes the pixel value of the reference image at the corresponding position; *m* and *n* represent the height and width of the image, respectively.

#### 2.5.2. Structural Similarity Index (SSIM)

The SSIM is adopted to evaluate structural quality by measuring the similarity of luminance, contrast, and structural patterns between the generated and GT images. A higher SSIM value indicates better preservation of spatial structures and contextual information, such as building boundaries, road layouts, and other geometric features in remote sensing images.

(14) $$\mathrm{SSIM}\left(x,y\right)=\frac{\left(2\mu_{x}\mu_{y}+C_{1}\right)\left(2\sigma_{xy}+C_{2}\right)}{\left(\mu_{x}^{2}+\mu_{y}^{2}+C_{1}\right)\left(\sigma_{x}^{2}+\sigma_{y}^{2}+C_{2}\right)}$$

where, *x* and *y* denote local patches extracted from the reconstructed image and the GT image, respectively. $\mu_{x}$ and $\mu_{y}$ are the local means of *x* and *y*; $\sigma_{x}^{2}$ and $\sigma_{y}^{2}$ are the corresponding variances; $\sigma_{xy}$ is the covariance between the two patches; and $C_{1}$ and $C_{2}$ are stability constants used to prevent the denominator from approaching zero.

#### 2.5.3. Learned Perceptual Image Patch Similarity (LPIPS)

LPIPS is introduced to assess perceptual similarity by comparing deep feature representations extracted from neural networks. A lower LPIPS value indicates that the generated image is closer to the GT image in terms of human-perceived visual characteristics, including more natural textures, smoother variations in vegetation-covered areas, and more realistic material representations on building surfaces.

(15) $$\mathrm{LPIPS}=\sum_{l}\frac{1}{H_{l}W_{l}}\sum_{h,w}{∥w_{l}⊙\left({\hat{y}}_{hw}^{l}-{\hat{y}}_{0hw}^{l}\right)∥}_{2}^{2}$$

where *x* denotes the reconstructed image and $x_{0}$ denotes the GT image; *l* indexes the *l*-th feature layer of a neural network; $H_{l}$ and $W_{l}$ represent the height and width of the feature map at that layer; and $w_{l}$ denotes learnable channel-wise weights to emphasize the relative importance of different feature channels. ${\hat{y}}_{hw}^{l}$ represents the normalized feature vectors at spatial position (h,w) in the *l*-th layer of a pretrained network, extracted from the reconstructed image and reference image. The feature difference is weighted channel-wise by $w_{l}$, so that discrepancies across different channels are scaled according to their learned importance.

These three metrics provide complementary evaluations of reconstruction performance: PSNR reflects pixel-level fidelity, SSIM measures structural consistency, and LPIPS captures perceptual similarity, together providing a more comprehensive assessment of super-resolution reconstruction quality.

## 3. Experiments and Results

### 3.1. Dataset Construction

In order to evaluate the effectiveness and generalizability of our method, we employ two complementary datasets: one is a synthetically generated dataset that is constructed by us with simulated thin cloud haze effects and additive noise, and the other is the public thin cloud dataset CUHK-CR1 [37].

#### 3.1.1. Our Dataset Construction

We selected 3900 high-resolution pre-disaster images from the public dataset xBD to construct our dataset for the SR task with cloud removal and denoising, as shown in Table 1 [40]. The selected images had a size of 1024×1024 pixels with spatial resolutions ranging from 0.3 m to 0.5 m, and covered diverse geographic regions, dense building distributions, and various land cover categories, exhibiting diverse spatial structural characteristics.

We designed synthetic cloud layers based on parameterized rules and overlaid them onto the original images using an alpha blending strategy to simulate the cloud haze occlusion commonly observed in remote sensing imagery [41]. Moreover, we simulated four representative cloud types: thin clouds, thick clouds, cirrus clouds, and stratus clouds. In scenes affected solely by cloud occlusion, each cloud type was sampled with equal probability. Each cloud layer was governed by a set of parameters, including optical thickness, cloud coverage ratio, spatial scale, streak morphology, and edge softness, which collectively determine the opacity, spatial distribution, textural characteristics, and boundary smoothness. This procedure generated synthetic cloud images with diverse coverage levels and realistic illumination variations. During fusion, alpha blending was applied as follows:

(16) $$C_{\mathrm{output}}=\alpha C_{\mathrm{cloud}}+\left(1-\alpha \right)C_{\mathrm{clear}}$$

where $C_{\mathrm{cloud}}$ denotes the synthetic cloud layer, $C_{\mathrm{clear}}$ represents the original remote sensing image, and α is the transparency coefficient controlling the intensity of cloud occlusion. This fusion strategy ensured that cloud-covered regions displayed brightness, color, and contrast characteristics consistent with real clouds in remote sensing images. Through this synthesis procedure, the generated images closely resembled real remote sensing scenes in terms of spatial distribution, illumination variations, and overall visual appearance, providing a realistic dataset for cloud haze removal tasks.

Moreover, to simulate Gaussian noise commonly encountered during sensor imaging and signal transmission, we randomly selected original images and injected multi-level noise. The noise was categorized into three intensity levels—mild, moderate, and severe—corresponding to standard deviation ranges of σ∈[4,10],[11,20], and [21,30]. To ensure realism and diversity in the data distribution, we adopted stratified and log-uniform sampling strategies, which increased the frequency of low-intensity noise samples. These strategies produced a more realistic noise distribution.

Furthermore, to improve realism and better simulate real-world interference, we randomly added cloud and fog as well as noise interference to some of the data.

Finally, we cropped both the synthesized degraded images and corresponding HR images from 1024×1024 to 256×256 pixels to reduce the model learning difficulty and minimize GPU resource consumption. The synthesized degraded images were then downsampled to 64×64 pixels using bicubic interpolation to serve as LR images. In addition, we divided them into training, validation, and test sets in an 8:1:1 ratio, and our dataset contained 3120 training pairs, 390 validation pairs, and 390 test pairs.

#### 3.1.2. CUHK-CR1

The CUHK-CR1 dataset is built upon high-resolution imagery acquired by the Jilin-1 satellite [37]. It comprises 668 pairs of images with 0.5 m resolution and 512×512 pixels, and the acquired time gap is 17 days between the cloudy image and its corresponding clear image. The scenes predominantly feature residential buildings, road networks, and vegetation. The dataset is particularly suitable for evaluating super-resolution methods in terms of boundary sharpness and textural fidelity.

To construct the benchmark, we first cropped each 512×512 image into non-overlapping 256×256 patches. And the corresponding clear (cloud-free) images serve as ground-truth high-resolution (HR) samples. Subsequently, the cloudy images were downsampled to 64×64 via bicubic interpolation to generate corresponding low-resolution (LR) counterparts. The final dataset contains 2572 image pairs. Specifically, the training set consists of 2138 pairs, while the validation and test sets each contain 217 pairs.

### 3.2. Evaluation Details

All experiments were implemented on a single NVIDIA A100 GPU with 40 GB of memory. DiTNet adopted 16-head multi-head self-attention and set the channel width to 768. Our method was trained using the AdamW optimizer with a perceptual loss function, and the number of sampling steps was set to 50. The initial learning rate was set to $1\times 10^{-4}$ and was dynamically adjusted following a cosine decay schedule.

### 3.3. Evaluation Results

#### 3.3.1. Ablation Experiments

In order to validate the effectiveness of the MSIS module and DiTNet in our FlowT-SR, a series of ablation experiments were conducted on our dataset. The quantitative results are presented in Table 2. The “baseline” was the original flow matching method based on U-Net [33]. The “base+MSIS” was obtained by integrating the MSIS module into the baseline. The “base+DiTNet4” replaced the original U-Net in the baseline configuration with a 4-layer DiTNet, while the “base+DiTNet8”, “base+DiTNet10” and “base+DiTNet12” replaced the original U-Net with an 8-layer, 10-layer and 12-layer DiTNet, respectively.

Table 2 presents the ablation results of different components and configurations in FlowT-SR (“base+MSIS+DiTNet10”). The baseline model achieves a PSNR of 27.76, an SSIM of 0.69, and an LPIPS of 0.142. After integrating the MSIS module, the performance improves to 27.88, 0.71, and 0.135, respectively, demonstrating that MSIS effectively enhances feature representation by capturing thin-cloud-haze-related spatial distributions and channel-wise spectral variations, thereby improving artifact suppression and detail preservation. In particular, the MSIS module performs well in extracting features from uniformly distributed interference, such as thin clouds and stratus clouds. However, its effectiveness is limited for thick clouds and cirrus clouds.

We further investigate the influence of different DiTNet depths on reconstruction performance. As the DiT depth increases from 4 to 10 layers, the model achieves consistent improvements. This indicates that a deeper transformer structure provides stronger capability for modeling long-range dependencies and complex spatial correlations in remote sensing images, leading to more accurate vector field prediction. However, further increasing the depth to 12 layers does not provide additional improvement when used alone, suggesting that an excessively deep transformer may introduce redundant representation capacity under the current reconstruction setting.

By combining MSIS with different DiTNet configurations, the complementary benefits of the two modules can be observed. Specifically, the FlowT-SR configuration with DiTNet10 achieves the best overall performance, reaching 29.63 PSNR, 0.76 SSIM, and 0.125 LPIPS.

The results reported in Table 2 are obtained using 50 sampling steps. When increasing the number of sampling steps to 100, the performance metrics become 29.64, 0.75, and 0.123 for PSNR, SSIM, and LPIPS, respectively. The marginal improvement indicates that further increasing the sampling steps does not lead to significant performance gains, demonstrating that FlowT-SR can achieve high-quality reconstruction with a relatively small number of sampling steps.

#### 3.3.2. Comparison Experiments

This study compares our FlowT-SR with five state-of-the-art SR methods, including SR3 [26], EDiffSR [27], StableSR [28], DiT4SR [30], and SfmSR [34]. These methods represent mainstream approaches for image SR. Specifically, EDiffSR, StableSR and SR3 are diffusion-based SR methods, SfmSR is a flow-matching-based method, and DiT4SR is a diffusion–Transformer-based method. In terms of network architecture, EDiffSR, StableSR, and SR3 utilize U-Net-based architectures for prediction. DiT4SR and SfmSR rely on a transformer-based architecture with an attention mechanism. We retrained all comparative methods on our dataset according to their official implementations.

The quantitative comparison results are presented in Table 3. Our FlowT-SR achieved the best PSNR and SSIM scores while still maintaining a competitive LPIPS score, indicating the best balance between perceptual quality and fidelity. Moreover, compared with SfmSR, FlowT-SR achieves consistent improvements in PSNR and SSIM, demonstrating the effectiveness of the proposed architectural enhancements. Furthermore, FlowT-SR consistently outperforms diffusion-based methods such as SR3 and StableSR across all metrics, highlighting its superiority in joint SR, dehazing, and denoising tasks. In addition, the experimental results on our dataset are substantially superior to those obtained on the CUHK-CR1 dataset. This performance gap primarily stems from a non-negligible acquisition time gap between the cloudy image and the corresponding reference image (cloud-free) in the CUHK-CR1 dataset, which results in considerable spectral differences between the paired image samples of this public dataset. In summary, these experimental results demonstrate that our FlowT-SR effectively restores fine details with perceptual realism while preserving structural consistency. This makes it particularly suitable for dehazing and denoising in remote sensing image SR tasks.

**Figure 5:**
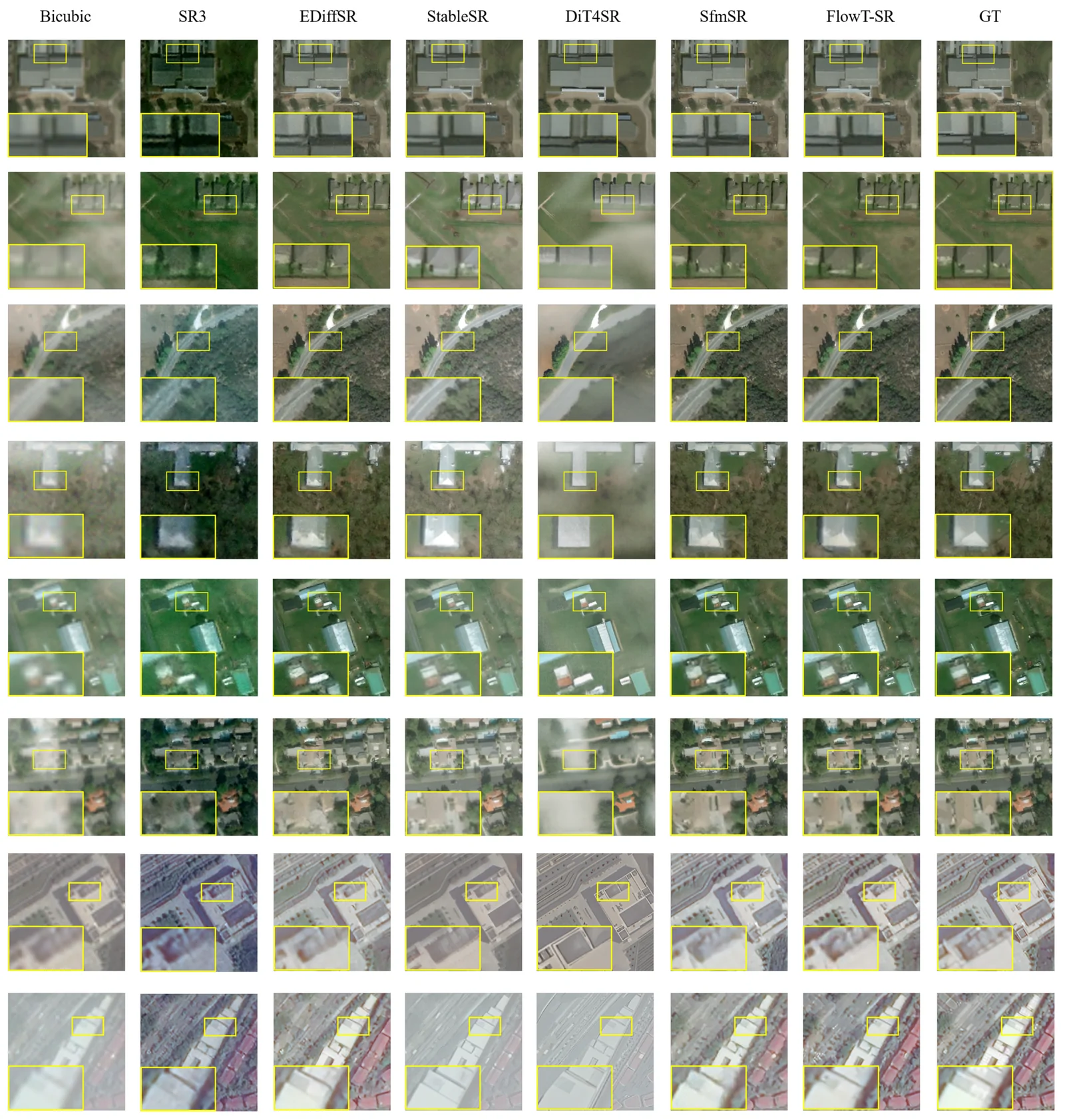
×16 visual comparison with state-of-the-art super-resolution (SR) models. Rows 1 to 6 are selected from our constructed dataset; rows 1 and 2 show images affected only by noise, rows 3 to 5 show images affected only by cloud haze, and row 6 shows images affected by both noise and haze. Rows 7 and 8 show images affected by thin cloud in CUHK-CR1.

We conducted a visual comparison with all comparative models. From Figure 5 (the yellow boxes highlight key enlarged regions for detailed inspection), we found that FlowT-SR still outperforms state-of-the-art SR methods in visual quality.

Shown in rows 1 and 2 of Figure 5, EDiffSR, SR3, and SfmSR exhibited limited capability in boundary reconstruction with noticeable blurring and discontinuities. In contrast, StableSR and DiT4SR were able to reconstruct relatively straight structural boundaries. However, these methods typically relied on large-scale pretrained generative models for initialization and were subsequently fine-tuned. By comparison, FlowT-SR was trained from scratch without pretrained weights and still produced clear and continuous structural details, demonstrating stronger capability in both noise suppression and structural reconstruction.

Rows 3–5 and 7–8 in Figure 5 present the super-resolution (SR) results under cloud-induced degradation. In this case, SR3 showed severe distortion, with reconstructed content deviating significantly from the ground-truth distribution. EDiffSR and SfmSR partly removed the influence of cloud haze, but slight noise and blurred details still remained in the reconstructed regions. Although StableSR performed relatively well in boundary reconstruction, its cloud haze removal capability was limited. This led to generated results with overly high brightness and insufficient contrast. DiT4SR struggled to recover meaningful texture information under cloud haze occlusion, resulting in noticeable artifacts and missing local details. In contrast, FlowT-SR delivered superior structural consistency and richer texture details, demonstrating its capacity to effectively remove cloud haze interference while preserving building boundaries.

The scenario in row 6 of Figure 5 is more challenging, as it is affected by both noise and cloud haze. SR3 generated noticeable noise artifacts and fragmented structures. EDiffSR reduced some degradation effects but still showed limited noise suppression, with slight boundary shifts and irregular structures in the reconstructed regions. StableSR produced relatively regular boundaries but introduced more cloud artifacts and generally high contrast. DiT4SR struggled to generate coherent and reliable details under combined noise and cloud haze interference. SfmSR partially mitigated the degradation effects, but its ability to reconstruct the structural continuity remained insufficient. In contrast, FlowT-SR effectively suppressed noise and removed cloud haze interference under complex degradation conditions while maintaining structural continuity and recovering more natural and reliable texture details. These results highlight the effectiveness of our MSIS module in mitigating cloud haze and noise interference in LR images, demonstrating the robustness and stability of FlowT-SR in complex remote sensing scenarios.

## 4. Conclusions

In this paper, we propose a novel super-resolution reconstruction framework based on flow matching and a diffusion transformer, named FlowT-SR, for optical remote sensing image SR with joint cloud haze and noise removal. Unlike conventional approaches that address super-resolution, haze removal, and denoising as separate tasks, FlowT-SR integrates these degradations into a unified generative reconstruction framework, enabling more faithful recovery of fine textures, structural details, and spectral information. Specifically, FlowT-SR constructs a conditional probability path via optimal transport displacement interpolation to constrain the image generation trajectory, which can be approximated as a linear transformation, and enables stable and high-quality reconstruction with fewer generation steps. Moreover, we introduce the MSIS module that extracts both channel-wise and spatial features to capture thin cloud haze positional information from multiple scales and perspectives while enhancing spectral signatures in LR images. Furthermore, we design DiTNet based on a diffusion transformer to predict the vector field, improving modeling of global spatial dependencies in remote sensing images. This boosts structural consistency in the generated images and reduces false edges and distorted details. Extensive experiments demonstrate that FlowT-SR achieves state-of-the-art performance across PSNR, SSIM, and LPIPS metrics, with reconstructed images exhibiting more coherent structural layouts, sharper texture details, and better-preserved spectral and geometric information. However, the performance of FlowT-SR may still be influenced by more challenging real-world conditions, such as severe cloud haze degradation, complex spatial variations, and domain differences, which require further investigation.

In future work, we will further optimize the FlowT-SR architecture to improve computational efficiency and reduce model complexity while maintaining reconstruction performance, making it more suitable for practical deployment. We also plan to extend the proposed framework to more challenging remote sensing scenarios, including post-disaster image super-resolution and reconstruction under thick cloud degradation with complex spatial structures and varying characteristics, thereby providing more reliable image data to support disaster assessment and emergency response.

## Figures and Tables

**Table 1:** The selected images from xBD.

Disaster Event Name	Directory Location and Image Number in xBD	No. of Images
Mexico City Earthquake	Hold/train/test all number	193
Midwest US Floods	Hold/train/test all number	445
Santa Rosa Wildfires	Hold/train/test all number	377
Hurricane Michael	Hold/train/test all number	550
Hurricane Florence	Hold/train/test all number	546
Guatemala Fuego Volcano Eruption	Hold/train/test all number	28
Sunda Strait Tsunami	Tier3 all number	105
Tuscaloosa, AL Tornado	Tier3 all number	288
Portugal Wildfires	Tier3 all number	957
Pinery Fire	Tier3 image number ≤ 1555	411
Total		3900

**Table 2:** Quantitative results of ablation experiments. The best performance is shown in bold. Arrow direction denotes whether higher or lower values of the metric are preferable.

Methods	Our Dataset		
	PSNR ↑	SSIM ↑	LPIPS ↓
Baseline	27.76	0.69	0.142
Base+MSIS	27.88	0.71	0.135
Base+DiTNet4	27.25	0.71	0.141
Base+DiTNet8	28.44	0.74	0.127
Base+DiTNet10	29.10	0.75	0.127
Base+DiTNet12	29.19	0.74	0.122
Base+MSIS+DiTNet8	29.23	0.75	0.126
Base+MSIS+DiTNet12	29.45	0.75	**0.115**
FlowT-SR	**29.63**	**0.76**	0.125

**Table 3:** Quantitative comparison with state-of-the-art SR models on our test set and the CUHK-CR1 dataset in terms of PSNR, SSIM, LPIPS, FLOPs(G), and Parameters(M). For each metric, the best performance is highlighted in bold. The best performance is shown in bold. Arrow direction denotes whether higher or lower values of the metric are preferable.

Methods	FLOPs (G)	Parameters (M)	Our Dataset			CUHK-CR1		
			PSNR ↑	SSIM ↑	LPIPS ↓	PSNR ↑	SSIM ↑	LPIPS ↓
SR3	155.24	155.33	19.21	0.59	0.203	19.26	0.59	0.18
EDiffSR	**46.25**	20.40	28.16	0.68	**0.121**	23.19	0.63	**0.13**
StableSR	7534.43	152.67	22.86	0.74	0.164	20.01	0.64	0.43
DiT4SR	237.25	952.62	20.30	0.60	0.363	19.40	0.46	0.45
SfmSR	232.283	**17.113**	28.52	0.72	0.127	23.57	0.65	0.22
FlowT-SR	291.048	109.62	**29.63**	**0.76**	0.125	**24.73**	**0.67**	0.15

## Data Availability

The public xBD and CUHK-CR1 datasets are available from their original providers. The data generated and analyzed during the current study are available from the corresponding author upon reasonable request.

## Abbreviations

The following abbreviations are used in this manuscript:

SR	Super-resolution
HR	High resolution
LR	Low resolution
GT	Ground truth
MSIS	Multi-scale interference suppression
DiT	Diffusion transformer
DiTNet	Diffusion transformer network
ODE	Ordinary differential equation
PSNR	Peak signal-to-noise ratio
SSIM	Structural similarity index
LPIPS	Learned perceptual image patch similarity

## References

[1] Drusch M., Del Bello U., Carlier S., Colin O., Fernandez V., Gascon F., Hoersch B., Isola C., Laberinti P., Martimort P. (2012). Sentinel-2: ESA’s optical high-resolution mission for GMES operational services. Remote Sens. Environ..

[2] Han J., Zhang D., Cheng G., Guo L., Ren J. (2014). Object detection in optical remote sensing images based on weakly supervised learning and high-level feature learning. IEEE Trans. Geosci. Remote Sens..

[3] Pan Z., Yu J., Huang H., Bao S., Zhang J. (2013). Super-resolution based on compressive sensing and structural self-similarity for remote sensing images. IEEE Trans. Geosci. Remote Sens..

[4] Yang D., Li Z., Xia Y., Chen Z. (2015). Remote sensing image super-resolution: Challenges and approaches. Proceedings of the 2015 IEEE International Conference on Digital Signal Processing (DSP).

[5] De Keukelaere L., Sterckx S., Adriaensen S., Knaeps E., Reusen I., Giardino C., Bresciani M., Hunter P., Neil C., Van der Zande D. (2018). Atmospheric correction of Landsat-8/OLI and Sentinel-2/MSI data using iCOR algorithm: Validation for coastal and inland waters. Eur. J. Remote Sens..

[6] Li G., Li J., Chen G., Wang Z., Jin S., Ding C., Zhang W. (2023). Delving deeper into image dehazing: A survey. IEEE Access.

[7] Wang P., Bayram B., Sertel E. (2022). A comprehensive review on deep learning based remote sensing image super-resolution methods. Earth-Sci. Rev..

[8] Wang X., Sun L., Chehri A., Jeon G. (2023). A review of GAN-based super-resolution reconstruction for optical remote sensing images. Remote Sens..

[9] Wang C., Sun W. (2025). Semantic guided large scale factor remote sensing image super-resolution with generative diffusion prior. Isprs J. Photogramm. Remote Sens..

[10] Aybar C., Contreras J., Donike S., Gonzales G., Fernandez C., Rosas J., Ochoa P., Moya K., Rudnick K., Heyn L. (2026). A radiometrically and spatially consistent super-resolution framework for Sentinel-2. Remote Sens. Environ..

[11] Tian C., Xu Y., Zuo W., Zhang B., Fei L., Lin C.W. (2020). Coarse-to-fine CNN for image super-resolution. IEEE Trans. Multimed..

[12] Dong R., Zhang L., Fu H. (2021). RRSGAN: Reference-based super-resolution for remote sensing image. IEEE Trans. Geosci. Remote Sens..

[13] Jia S., Wang Z., Li Q., Jia X., Xu M. (2022). Multiattention generative adversarial network for remote sensing image super-resolution. IEEE Trans. Geosci. Remote Sens..

[14] Zhang Y., Wei S., Sun Y., Shen J., Yang Z., Yan J. (2025). EESAGAN: Edge-Enhanced and Structure-Aware GAN for Remote Sensing Image Super-Resolution. IEEE J. Sel. Top. Appl. Earth Obs. Remote Sens..

[15] Lugmayr A., Danelljan M., Van Gool L., Timofte R. (2020). SRFlow: Learning the super-resolution space with normalizing flow. Proceedings of the European Conference on Computer Vision.

[16] Yao J.E., Tsao L.Y., Lo Y.C., Tseng R., Chang C.C., Lee C.Y. (2023). Local implicit normalizing flow for arbitrary-scale image super-resolution. Proceedings of the IEEE/CVF Conference on Computer Vision and Pattern Recognition.

[17] Song C., Li S., Li F.W., Yang B. (2025). WDFSR: Normalizing flow based on the wavelet-domain for super-resolution. Comput. Vis. Media.

[18] Moser B.B., Shanbhag A.S., Raue F., Palacio S., Frolov S., Dengel A. (2024). Diffusion models, image super-resolution, and everything: A survey. IEEE Trans. Neural Netw. Learn. Syst..

[19] Sun H., Zou J., Chen W., Han X., Li H., Zhang Y. (2025). HyperFlow: Conditional Normalizing Flow-Guided Spectral Super-Resolution With Learnable Sampler. IEEE Trans. Geosci. Remote Sens..

[20] Liang J., Cao J., Sun G., Zhang K., Van Gool L., Timofte R. (2021). Swinir: Image restoration using swin transformer. Proceedings of the IEEE/CVF International Conference on Computer Vision.

[21] Shi Y., Chen N., Pu Y., Zhang J., Yao L. (2024). SwinIBSR: Towards real-world infrared image super-resolution. Infrared Phys. Technol..

[22] Zhang J., Tu Y. (2025). SwinFR: Combining SwinIR and fast fourier for super-resolution reconstruction of remote sensing images. Digit. Signal Process..

[23] Dharejo F.A., Ganapathi I.I., Zawish M., Alawode B., Alathbah M., Werghi N., Javed S. (2024). SwinWave-SR: Multi-scale lightweight underwater image super-resolution. Inf. Fusion.

[24] Zhang X., Zhang Y., Yu F. (2024). HiT-SR: Hierarchical transformer for efficient image super-resolution. Proceedings of the European Conference on Computer Vision.

[25] Zeraatkar E., Faroughi S., Tešić J. (2025). Visir: Vision transformer single image reconstruction method for earth system models. arXiv.

[26] Saharia C., Ho J., Chan W., Salimans T., Fleet D.J., Norouzi M. (2022). Image super-resolution via iterative refinement. IEEE Trans. Pattern Anal. Mach. Intell..

[27] Xiao Y., Yuan Q., Jiang K., He J., Jin X., Zhang L. (2023). EDiffSR: An efficient diffusion probabilistic model for remote sensing image super-resolution. IEEE Trans. Geosci. Remote Sens..

[28] Wang J., Yue Z., Zhou S., Chan K.C., Loy C.C. (2024). Exploiting diffusion prior for real-world image super-resolution. Int. J. Comput. Vis..

[29] Miao R., Yang K., Zhou K., Song J., Fu S., Liu C., Wang Y. (2025). Research on cross-sensor remote sensing image super-resolution method based on diffusion models. IEEE J. Sel. Top. Appl. Earth Obs. Remote Sens..

[30] Duan Z.P., Zhang J., Jin X., Zhang Z., Xiong Z., Zou D., Ren J.S., Guo C., Li C. (2025). DiT4SR: Taming diffusion transformer for real-world image super-resolution. Proceedings of the IEEE/CVF International Conference on Computer Vision.

[31] Dhariwal P., Nichol A. (2021). Diffusion models beat GANs on image synthesis. Adv. Neural Inf. Process. Syst..

[32] Yang S., Ebrahimian H., Zhang Z., Sun W. (2026). Deep learning for single-image super-resolution in remote sensing: A review. Int. J. Remote Sens..

[33] Lipman Y., Chen R.T., Ben-Hamu H., Nickel M., Le M. (2022). Flow matching for generative modeling. arXiv.

[34] Gong Z., Yi F., Guan L., Dong C., Zeng H. (2025). Semantic-Guided Flow Matching for Fast and Accurate Remote Sensing Image Super-Resolution. IEEE Trans. Geosci. Remote Sens..

[35] Feng X., Zhang W., Su X., Li J. (2021). Optical remote sensing image denoising and super-resolution reconstructing using optimized generative network in wavelet transform domain. Remote Sens..

[36] Jozdani S., Chen D., Pouliot D., Johnson B.A. (2022). A review and meta-analysis of generative adversarial networks and their applications in remote sensing. Int. J. Appl. Earth Obs. Geoinf..

[37] Sui J., Ma Y., Yang W., Liu X. (2024). Diffusion enhancement for cloud removal in ultra-resolution remote sensing imagery. arXiv.

[38] Wang Z., Bovik A.C., Sheikh H.R., Simoncelli E.P. (2004). Image quality assessment: From error visibility to structural similarity. IEEE Trans. Image Process..

[39] Zhang R., Isola P., Efros A.A., Shechtman E., Wang O. (2018). The unreasonable effectiveness of deep features as a perceptual metric. Proceedings of the IEEE Conference on Computer Vision and Pattern Recognition.

[40] Gupta R., Hosfelt R., Sajeev S., Patel N., Goodman B., Doshi J., Heim E., Choset H., Gaston M. (2019). xBD: A dataset for assessing building damage from satellite imagery. arXiv.

[41] Czerkawski M., Atkinson R., Michie C., Tachtatzis C. (2023). SatelliteCloudGenerator: Controllable cloud and shadow synthesis for multi-spectral optical satellite images. Remote Sens..

